# A comparison of Scansys and Sirius tomography in healthy eyes

**DOI:** 10.1186/s12886-024-03389-7

**Published:** 2024-03-27

**Authors:** Masoud Khorrami-Nejad, Mehdi Khodaparast, Ihsan Ali Abdulkadhim, Elham Azizi, Fatemeh Rashidi, Vahid Damanpak, Hesam Hashemian

**Affiliations:** 1 Translational Ophthalmology Research Center, Farabi Eye Hospital, Tehran University of Medical Sciences, Tehran, Iran; 2 School of Rehabilitation, Tehran University of Medical Sciences, Tehran, Iran; 3 Department of Optometry and Vision Sciences, University of Melbourne, Melbourne, Australia; 4 Farabi Eye Hospital, Tehran University of Medical Sciences, Kargar Street, Tehran, Iran

**Keywords:** Anterior segment, Scheimpflug imaging, Scansys, Sirius, Agreement, Interchangeability

## Abstract

**Purpose:**

To assess the level of agreement and evaluate the reliability of measurements between two Scheimpflug imaging modalities, Scansys (MediWorks, China) and Sirius (CSO, Italy), in quantifying the anterior segment parameters in healthy eyes.

**Methods:**

In a cross-sectional study, the right eyes of 38 healthy participants without any ocular or systemic diseases were examined. A range of anterior segment parameters including anterior and posterior flat and steep keratometry, central corneal thickness (CCT), thinnest corneal thickness (TCT), anterior chamber depth (ACD), anterior chamber angle (ACA), corneal volume, anterior chamber volume, and horizontal white to white diameter, derived from the sagittal curvature maps were measured. To evaluate the reliability of the measurements, intraclass correlation coefficient (ICC) and correlation coefficient were measured. Additionally, Bland-Altman plots were employed to examine the agreement in mean (bias line) and 95% limits of agreement between the two devices.

**Results:**

The mean age was 31.5 ± 6.9 (range: 19–47) years. The ICC indicated that the majority of anterior segment parameters had an excellent or good level of reliability, surpassing the threshold of 0.9. Nevertheless, CCT and ACA exhibited a moderate level of reliability, with ICC values of 0.794 and 0.728, respectively. The correlation analysis showed a strong correlation for all the variables tested. The Bland-Altman plots revealed that the bias line was near zero and the 95% limits of agreement were narrow for most variables, except for the anterior flat and steep keratometry, which were found to range from − 0.57 to 0.84 D and − 0.68 to 0.87 D, respectively.

**Conclusion:**

Scansys and Sirius devices can be effectively used interchangeably for the evaluation of most anterior segment parameters; however, for anterior corneal curvatures, CCT and ACA, their alternative use is not recommended.

## Introduction

Optical systems for anterior segment evaluation offer a noncontact approach that is highly convenient and rapid for both eye care professionals and patients [1, 2]. These systems have the capacity to acquire a multitude of anterior segment biometric measurements in a single comprehensive scan [3, 4]. Corneal tomography measurement is one of the most common diagnostic techniques in the detection of corneal ectatic diseases, particularly at earlier stages which can display anterior and posterior segment parameters, including curvature, elevation, and corneal thickness profile [5, 6]. 

Scheimpflug imaging is a popular and precise method for measuring the biometric and ocular surface characteristics of the anterior segment [7, 8]. Different tomography devices such as Galilei (Ziemer, Port, Switzerland), Sirius (Costruzione Strumenti Oftalmici, Florence, Italy), and Pentacam (Oculus, Wetzlar, Germany) are available as the most well-known devices for measuring the tomographic parameters [7, 9]. The Sirius tomography instrument has an analysis system for the anterior segment, combining Placido disc and Scheimpflug camera technology [10, 11]. The device provides a full analysis of the anterior segment parameters, corneal curvature, corneal thickness and corneal wave front map [10]. It uses visible light (blue light) with a wavelength of 475 nm to perform rotational 360° photography and collect 25 or 50 images of the anterior segment profile in approximately 5 s over a diameter of 12 mm. Another tomography device, with Scheimpflug technology, which has recently been released to the market, named as Scansys, utilizes a camera to measure the anterior segment parameters [12]. This device can measure more than 107,520 data points with 28 high resolution slit images of the both corneal surfaces in only one second. This device uses a slit-light source and the wavelength is 470 nm. It can generate multiple corneal topography maps, including corneal curvature, corneal thickness, and corneal elevation. The horizontal and vertical measurement ranges over the corneal surface are up to 14 mm and 10 mm, respectively. Scansys can track the involuntary micro eye movements and decrease the motion error by correcting eye movements through a software algorithm [7]. 

Before a new device can be used for routine clinical examinations, it is necessary to determine the reliability of its measurements and their agreement with already available and popular instruments. Several studies have validated the repeatability, precision, and agreement between different corneal tomography devices [11, 13–15]. However, very little work has been undertaken to evaluate the agreement between Scansys and other corneal tomography systems. Few evidence have been obtained in comparison of the Scansys with the gold standard Pentacam HR and they showed an excellent inter-observer reproducibility and intra-observer repeatability for corneal thickness measurements, corneal volume and anterior corneal curvature between the two devices [15, 16]. However, there is still no evidence on comparison of this device with the Sirius.

Hence, the primary objective of this current investigation is to undertake a comparative analysis of a range of anterior segment parameters between the Scansys and Sirius devices and investigate the interchangeability of the devices in measuring the parameters examined.

## Patients and methods

### Study design and ethical considerations

This cross-sectional analysis was carried out on 38 right eyes of 38 healthy adult participants over 18 years of age. The participants were recruited during routine ophthalmic examinations at Farabi Eye Hospital in Tehran, Iran. The research protocol received ethical approval from the institutional review board at Tehran University of Medical Sciences (IR.TUMS.FNM.REC.1402.053) and adhered to the Declaration of Helsinki principles. The protocols of the study was explained for the participants and then informed written consent was obtained from all participants.

The exclusion criteria contained any prior history of ocular surgery or trauma, presence of corneal diseases, current use of gas-permeable or mini-scleral contact lenses, dry eye disease, acute or progressive ocular comorbidities, and systemic diseases with ocular involvement. Participants who utilized soft contact lenses were required to discontinue lens wear for at least two weeks prior to the study examinations. These exclusion criteria were set to minimize potential confounding ocular anatomical and physiological factors that could interfere with obtaining the normal anterior segment measurements. The included patients, therefore, contained those without a history of ocular or systemic diseases.

### Examinations

Study participants underwent routine ophthalmic evaluations, including assessment of uncorrected and best-corrected distance visual acuity, refractive error and slit lamp biomicroscopic examination. Subsequently, each participant received imaging of the anterior segment using two devices: the Scansys Anterior Segment 3D Analyzer (MediWorks, Shanghai, China) followed by the Sirius Corneal Topography system (Costruzione Strumenti Oftalmici, Florence, Italy). Examinations occurred in a darkened room to avoid confounding effects of the external light on pupils. An experienced optometrist performed all measurements over approximately 20 min, with a 15-minute interval between repeated scans on the two devices. To control for diurnal variations in ocular parameters, imaging was conducted between 10:00 am to 13:00 pm.

### Outcome measures

The measured parameters from both devices included flat and steep meridians of the anterior and posterior keratometry reading, central corneal thickness (CCT), thinnest corneal thickness (TCT), anterior chamber depth (ACD), anterior chamber angle (ACA), corneal volume, anterior chamber volume, and HWTW diameter, derived from the sagittal curvature maps. The ACA was calculated using tomography images obtained by the Scheimpflug camera in both devices. The images were then analyzed using computer software, and the ACA was presented in degrees.

### Statistical analysis

A priori sample size calculation using G*Power 3.1 was conducted. Based on α = 0.05, power = 0.8, and an effect size of 0.5 for two-tailed t-tests, the estimated required sample size was 34 participants. The collected data were analyzed using the SPSS software (IBM Inc., Chicago, USA). The normality of data distribution was confirmed using the Shapiro-Wilk test. For normally distributed parameters, differences between devices were compared using independent samples t-tests. For non-normal data, the non-parametric Mann-Whitney U test was utilized for comparisons. A p-value < 0.05 was considered statistically significant. The correlation and agreement between Scansys and Sirius devices were analyzed for all the measured variables using a range of statistical analyses. Intraclass correlation coefficients (ICC) evaluated reproducibility between devices. Regression analysis determined mathematical conversion factors between devices. Bland-Altman plots visualized agreement with 95% confidence intervals or 95% limits of agreement. Vector analysis was performed to report the anterior and posterior corneal astigmatism measured by the two devices. A p-value < 0.05 was considered statistically significant.

## Results

This study involved examination of 38 right eyes from a cohort of 38 patients, with an average age of 31.5 ± 6.9 years (ranging from 19 to 47 years). The participant group comprised 16 males (42.1%) and 22 females (57.9%).

The intraclass correlation coefficients (ICC) and correlation analysis for flat-K and steep-K values in both the anterior and posterior corneal surfaces exceeded 0.9, indicating excellent agreement between the two instruments. Detailed comparisons of keratometry measurements for both anterior and posterior corneal surfaces between the Sirius and Scansys instruments are provided in Table 1. The results indicate that for the anterior corneal surface, the mean flat-K values were 43.18 ± 1.59 D for Sirius and 43.04 ± 1.40 D for Scansys, revealing a statistically significant difference between the two devices (*P* = 0.026); however, this is not clinically meaningful. Regarding anterior steep-K values, no statistically significant difference was detected between Sirius and Scansys (*P* = 0.156).

**Table 1:** Comparison of keratometry of anterior and posterior corneal surfaces between Sirius (Costruzione Strumenti Oftalmici, Italy) and Scansys (MediWorks, Shanghai, China)

			Device	Minimum	Maximum	Mean ± SD	Correlation		ICC	Difference of mean ± SD*	95% CI of differences of means		*P*-value**
							r	*P*-value			***Lower***	***Upper***	
Anterior	Flat K (D)		Sirius	38.50	46.15	43.18 ± 1.59	0.979	< 0.001	0.985	0.14 ± 0.36	0.02	0.25	0.026
			Scansys	39.92	45.85	43.04 ± 1.40							
	Steep K (D)		Sirius	41.24	47.59	44.35 ± 1.48	0.964	< 0.001	0.980	0.09 ± 0.39	-0.04	0.22	0.156
			Scansys	40.78	47.53	44.26 ± 1.36							
	Astigmatism	J0	Sirius	-0.61	2.23	0.48 ± 0.58	0.957	< 0.001	0.978	0.00 ± 0.17	-0.06	0.05	0.965
			Scansys	-0.58	2.27	0.48 ± 0.56							
		J45	Sirius	-0.32	0.41	0.04 ± 0.18	0.855	< 0.001	0.902	-0.04 ± 0.13	-0.08	0.00	0.069
			Scansys	-0.41	0.63	0.08 ± 0.24							
Posterior	Flat K (D)		Sirius	-5.67	-6.47	-6.06 ± 0.19	0.956	< 0.001	0.977	-0.14 ± 0.06	-0.16	-0.12	< 0.001
			Scansys	-6.28	-5.59	-5.92 ± 0.20							
	Steep K (D)		Sirius	-6.05	-6.97	-6.46 ± 0.22	0.971	< 0.001	0.985	-0.21 ± 0.05	-0.22	-0.19	< 0.001
			Scansys	-6.73	-5.84	-6.25 ± 0.22							
	Astigmatism	J0	Sirius	-0.45	0.05	-0.19 ± 0.10	0.973	< 0.001	0.984	-0.03 ± 0.02	-0.04	-0.02	< 0.001
			Scansys	-0.42	0.02	-0.16 ± 0.09							
		J45	Sirius	-0.14	0.07	-0.03 ± 0.05	0.578	< 0.001	0.732	-0.03 ± 0.04	-0.05	-0.02	< 0.001
			Scansys	-0.08	0.12	0.01 ± 0.04							

*Differences of the mean values calculated by subtracting measurements of Sirius from Scansys

**Based on independent samples t-test

D: diopter, N: Number, ICC: intraclass correlation coefficient

For posterior flat-K, Sirius recorded − 6.06 ± 0.19 D and Scansys measured − 5.92 ± 0.20 D (*P* < 0.001) and for the posterior steep-K, it was − 6.46 ± 0.22 D for Sirius and − 6.25 ± 0.22 D for Scansys (*P* < 0.001). The vector analysis (J0, J45) was also conducted for the anterior and posterior corneal astigmatism, revealing excellent agreement (ICC > 0.9) and very strong correlation (*r* > 0.9) between devices in the anterior cornea as well as J0 in the posterior corneal surface. However, for J45 in posterior corneal astigmatism, there was good agreement (ICC = 0.732) and moderate correlation (*r* = 0.578) between devices, but the mean difference (-0.03 ± 0.04 D) was negligible (Table 1).

**Fig. 1:**
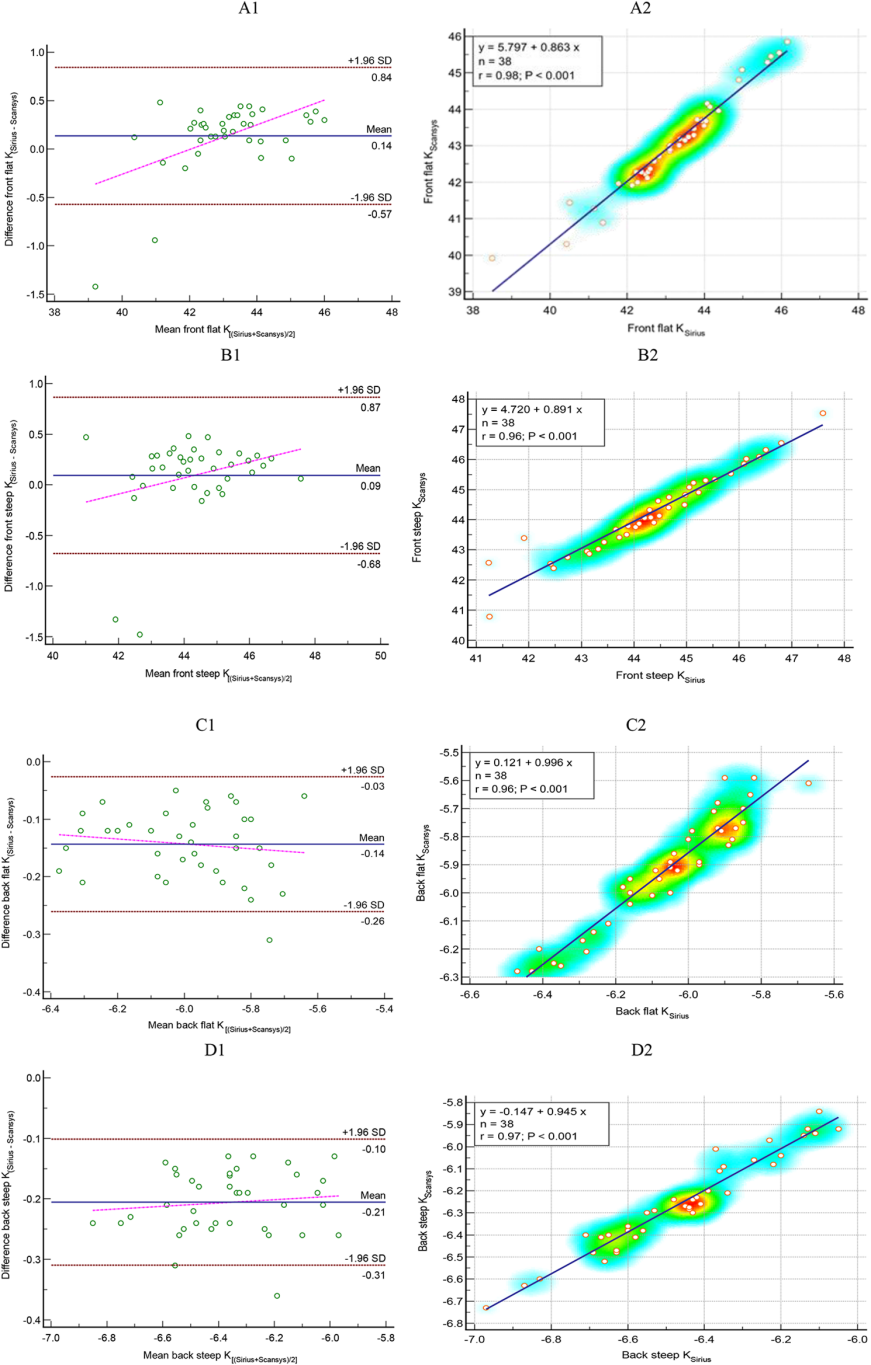
The left plots show Bland–Altman plots for A1- front flat keratometry (D), B1- front steep K (D), C1- back flat K (D) and D1- back steep K(D) comparing Sirius (Costruzione Strumenti Oftalmici, Florence, Italy) vs. Scansys (MediWorks, Shanghai, China). The mean difference, 95% limits of agreement and regression line are shown by blue solid line, red and pink dash-dotted lines, respectively. The right Scatter plots compare mean values of A2- front flat keratometry (D), B2- front steep K (D), C2- back flat K (D) and D2- back steep K (D) measured with Sirius (Costruzione Strumenti Oftalmici, Florence, Italy) and Scansys (MediWorks, Shanghai, China). The blue dash-dotted lines show the regression lines. K, keratometry

Figure 1 displays the Bland–Altman plot for flat K and steep K values in both the anterior and posterior corneal surfaces, providing a comparative analysis between the Sirius and Scansys devices. As plots illustrate there was relatively wide 95% limits of agreement for the anterior flat (-0.57 to 0.84 D) and steep-K (-0.68 to 0.87 D). However, for the posterior flat and steep-K, the limits of agreement was narrow.

**Fig. 2:**
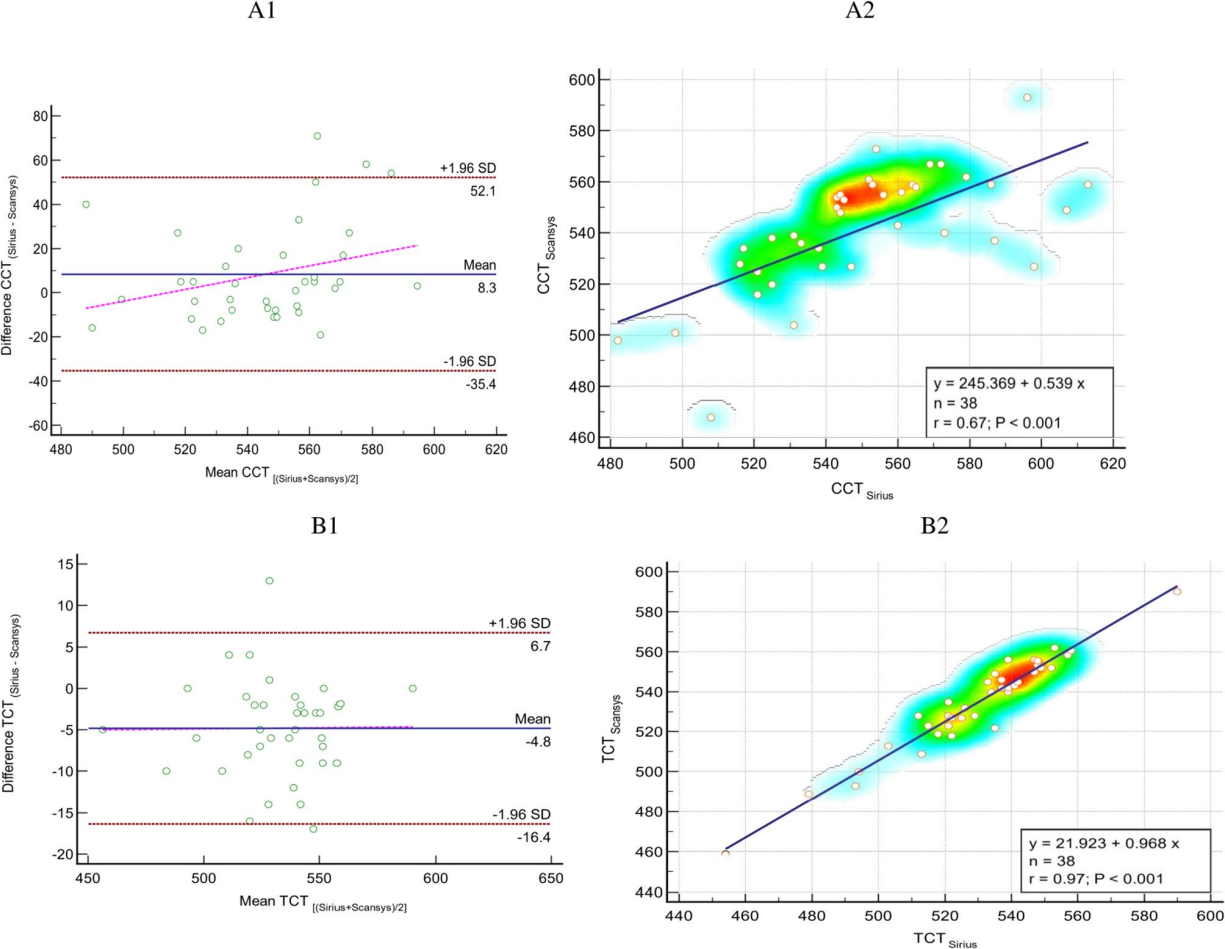
The left plots show Bland–Altman plots for A1- central corneal thickness (µm) and B1- thinnest corneal thickness (µm), comparing Sirius (Costruzione Strumenti Oftalmici, Florence, Italy) vs. Scansys (MediWorks, Shanghai, China). The mean difference, 95% limits of agreement and regression line are shown by blue solid line, red and pink dash-dotted lines, respectively. The right Scatter plots compare mean values of A2- central corneal thickness (µm) and B2- thinnest corneal thickness (µm) measured with Sirius (Costruzione Strumenti Oftalmici, Florence, Italy) and Scansys (MediWorks, Shanghai, China). The blue dash-dotted lines show the regression lines. K, keratometry; CCT, central corneal thickness; TCT, thinnest corneal thickness

**Fig. 3:**
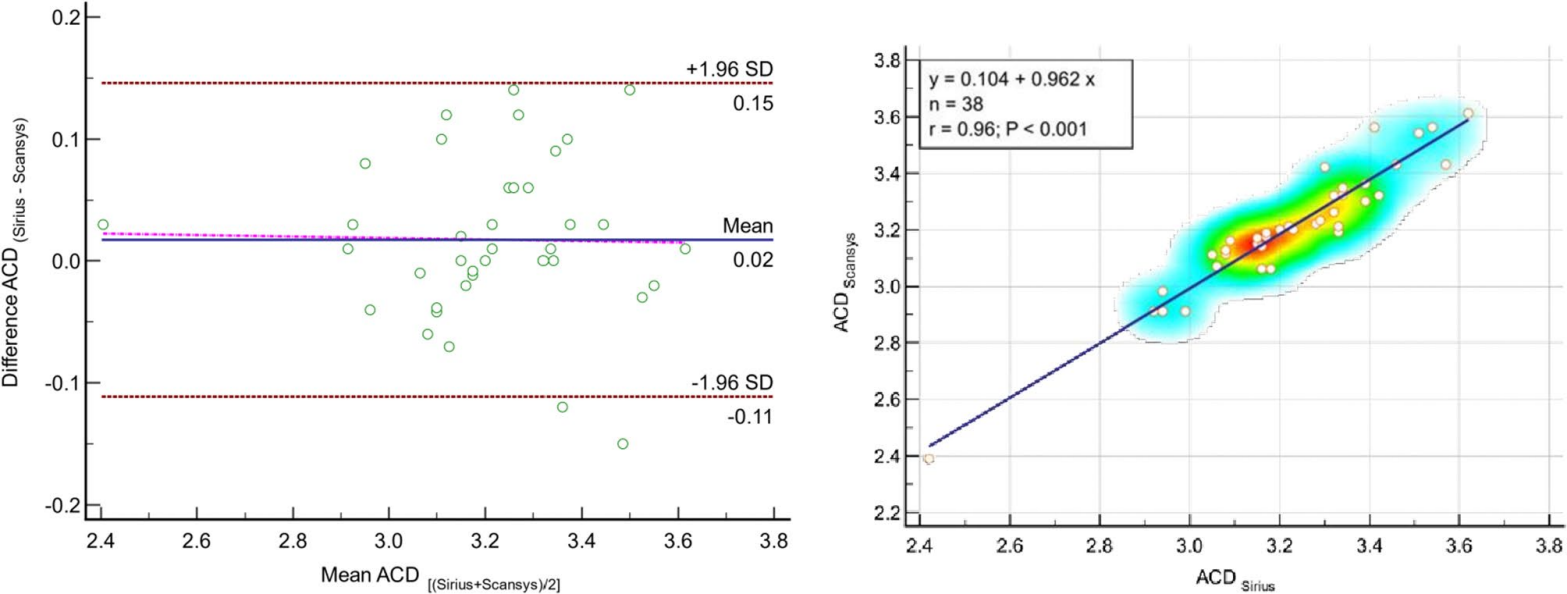
A1- Bland–Altman plot for anterior chamber depth (mm) comparing Sirius (Costruzione Strumenti Oftalmici, Florence, Italy) vs. Scansys (MediWorks, Shanghai, China). The mean difference, 95% limits of agreement and regression lines are shown by the blue solid line, red and pink dash-dotted lines, respectively. A2- Scatter plots to compare mean values of anterior chamber depth (mm) measured with Sirius (Costruzione Strumenti Oftalmici, Florence, Italy) and Scansys (MediWorks, Shanghai, China). The blue dash-dotted lines show the regression lines. ACD, anterior chamber depth

Table 2 provides a detailed comparison of pachymetry indexes, ACD, ACA, HWTW, corneal volume, and chamber volume between the Sirius and Scansys devices. The table illustrates the level of agreement between these devices, with ICC values exceeding 0.9 for all metrics, except for CCT (0.794), ACA (0.728), and chamber volume (0.884), which exhibited only moderate agreement. Furthermore, the table highlights statistically significant differences in mean values measured by each device, for all parameters listed in Table 2 (all *P* < 0.05), except for the ACD measurements (*P* = 0.111). Figures 2 and 3 display the Bland–Altman plots for CCT, TCT, and ACD, when comparing measurements obtained with the Sirius and Scansys devices.

**Table 2:** Comparison of pachymetric indexes, ACD, ACA, IOP sum, HWTW, corneal volume and chamber volume between Sirius (Costruzione Strumenti Oftalmici, Italy) vs. Scansys (MediWorks, Shanghai, China)

	Device	N	Minimum	Maximum	Mean ± SD	Correlation		ICC	Difference of mean ± SD*	95% CI of differences of means		*P*-value**
						r	P-value					
										Lower	Upper	
TCT (µm)	*Sirius*	38	454.00	590.00	529.68 ± 24.14	0.970	< 0.001	0.985	-4.82 ± 5.89	-6.75	-2.88	0.000
	*Scansys*	38	459.00	590.00	534.50 ± 24.08							
CCT (µm)	*Sirius*	38	482.00	613.00	549.89 ± 29.85	0.675	< 0.001	0.794	8.34 ± 22.33	1.00	15.68	0.027
	*Scansys*	38	468.00	593.00	541.55 ± 23.82							
ACD (mm)	*Sirius*	38	2.42	3.62	3.22 ± 0.22	0.957	< 0.001	0.978	0.02 ± 0.07	0.00	0.04	0.111
	*Scansys*	38	2.39	3.61	3.21 ± 0.22							
ACA (degree)	*Sirius*	38	33.00	55.00	46.92 ± 4.24	0.688	< 0.001	0.728	5.26 ± 3.14	4.23	6.30	< 0.001
	*Scansys*	38	33.00	45.00	41.66 ± 2.27							
HWTW (mm)	*Sirius*	38	11.24	12.86	12.17 ± 0.37	0.821	< 0.001	0.901	0.11 ± 0.22	0.04	0.18	0.003
	*Scansys*	38	11.06	12.70	12.06 ± 0.35							
Corneal volume (mm^3^)	*Sirius*	38	50.40	64.30	57.82 ± 2.48	0.881	< 0.001	0.937	-3.19 ± 1.22	-3.59	-2.78	< 0.001
	*Scansys*	38	53.71	67.91	61.01 ± 2.54							
Chamber volume (mm^3^)	*Sirius*	38	102.00	236.00	185.71 ± 23.85	0.795	< 0.001	0.884	20.47 ± 16.03	15.20	25.74	< 0.001
	*Scansys*	38	93.75	220.97	165.24 ± 25.82							

*Differences of the mean values calculated by subtracting measurements of Sirius HR from Scansys

**Based on independent samples t-test

N: Number, ICC: intraclass correlation coefficient, CCT: central corneal thickness, TCT: thinnest corneal thickness, ACD: anterior chamber depth, ACA: anterior chamber angle, HWTW: horizontal white-to-white

## Discussion

Scheimpflug imaging has become a widely accepted method for precise measurement of various biometric and ocular surface characteristics of the anterior segment [17]. This study was designed to assess the compatibility of two Scheimpflug-based imaging technologies, namely, Scansys and Sirius, in a cohort of healthy individuals, with the aim of providing a foundation for reliability of the Scansys measurements and its potential interchangeability with Scansys. Our findings indicated excellent reliability for most anterior segment parameters, as demonstrated by high intraclass correlation coefficients (ICCs) exceeding 0.9, except for the ACA (ICC = 0.728) and CCT (ICC = 0.794). Furthermore, the agreement between the two devices was excellent for the majority of metrics, including TCT, ACD, HWTW, and corneal volume, considering the correlation analysis. However, there was only moderate correlation for CCT, ACA, and chamber volume. The Bland-Altman analysis revealed that, in general, the measurements from both devices were in close agreement with minimal bias. The 95% limits of agreements were narrow and clinically insignificant for most parameters, except for the anterior flat and steep K with − 0.57 to 0.84 D and − 0.68 to 0.87 D, respectively. Consequently, these findings suggest that Scansys and Sirius are essentially interchangeable for most anterior segment measurements. But their interchangeability is not recommended for the CCT, ACA and anterior flat and steep keratometry.

Within the range of corneal parameters, two critical factors for assessing eligibility for refractive surgery are corneal thickness and corneal curvature [18]. The precision of these measurements plays a pivotal role in evaluating the potential risks associated with post-operative complications like corneal ectasia and keratectasia [19–21]. Several studies have delved into the agreement among Scheimpflug devices, including Sirius when measuring corneal curvature and pachymetry in healthy eyes [22–26]. In a prospective investigation, Anayol et al. [13] conducted a study to assess the concordance of three Scheimpflug-based instruments, namely Pentacam HR, Sirius, and Galilei, in quantifying anterior segment parameters in healthy individuals. Regarding CCT, their study identified a significant variation among the three devices (*P* < 0.001), with Galilei reporting higher measurements compared to Pentacam HR and Sirius. Moreover, Pentacam HR and Sirius instruments demonstrated better agreement with each other than with Galilei. Their findings also extended to TCT measurements, with Pentacam and Galilei differing by -13.93 μm and Sirius differing by -5.5 μm. As for corneal curvature, the study noted that Galilei and Sirius devices exhibited stronger agreement with each other than with Pentacam HR, in contrast to Wang et al.‘s [27] which indicated good agreement between Pentacam HR and Sirius in measuring the anterior corneal curvature. Anayol et al. [13] concluded that these three Scheimpflug-based systems should not be employed interchangeably for measuring corneal thickness and curvature data in healthy eyes. Nasser et al. [11] supported this conclusion when comparing corneal curvature measurements between Sirius and Pentacam HR.

However, there are few reports for the reliability and agreement of the new Scansys with other available Scheimpflug devices. Yu et al. [28] conducted a comprehensive examination to assess the reliability and concordance of pachymetry measurements at various corneal locations using Scansys and Pentacam HR. They concluded that there was excellent inter-observer reproducibility and intra- observer repeatability for the Scansys and the device could be used interchangeably with Pentacam HR for the corneal thickness measurements in the central region and in the 2 mm from central region. We compared Scansys with Sirius and found strong agreement between the devices for the TCT (ICC = 0.985) and corneal volume (ICC = 0.937); however, the there was a moderate agreement (ICC = 0.728) for the CCT. Therefore, their interchangeable use might not be suggested for the CCT. It is noteworthy that our investigation detected statistically higher values for TCT and corneal volume with Scansys compared to those obtained with the Sirius device. However, in terms of CCT, Scansys yielded significantly lower values than Sirius.

In terms of the corneal curvature data, Xu et al. [15] suggested that Scansys can be used interchangeably with Pentacam HR, only in measuring the anterior corneal curvature. In contrast, we found excellent agreement between Scansys and Sirius in measuring the anterior and posterior keratometry and corneal astigmatism; although, there was a wide 95% limits of agreement for the anterior flat and steep keratometry, which might underscore the agreement between the two devices in measuring the anterior keratometry. This result is in contrast with Xu et al.; although, they are not directly comparable as the devices used were not the same.

The ACD has been established as a crucial factor for preoperative evaluation in intraocular surgeries. Its significance cannot be overstated, as evidenced by several studies that compared it using various Scheimpflug corneal imaging devices [29, 30]. While some studies report that these devices are interchangeable and yield differences that fall within clinically acceptable levels, [14, 22, 25, 31] others, such as Anoyal et al., [13] have found significant differences in ACD findings. The authors issued a cautionary note regarding the interchangeable use of these instruments, highlighting the potential for miscalculations in intraocular lens (IOL) power due to substantial disparities in ACD measurements. In contrast, our present investigation unveiled a robust agreement in this variable between the Scansys and Sirius devices (ICC = 0.978). Furthermore, there were no statistically significant differences in ACD measurements recorded by the two devices (*P* = 0.111).

The measurement of HWTW diameter within the anterior segment holds importance in the calculation of refractive intraocular lens power [32]. Nevertheless, the assessment of agreement among various corneal topography devices has remained a relatively understudied area, yielding inconsistent outcomes [33, 34]. While certain investigations advocate for the interchangeability of corneal topography devices in measuring HWTW, [31] others take a more cautious manner [35–37]. In the domain of Scheimpflug-based imaging technologies, Domínguez-Vicent et al. [38] recently conducted an assessment of the agreement between Pentacam HR and Galilei, concerning HWTW measurements. Their findings indicated that due to the clinically significant wide 95% limit of agreement, these two Scheimpflug imaging systems should not be used interchangeably. In the present study, we observed that HWTW measurements exhibited a statistically significant difference, with Sirius measurements notably higher than those obtained with the Scansys device (*P* = 0.003). However, it is noteworthy that the ICC demonstrated excellent agreements between the two devices (ICC = 0.901).

This study encountered some limitations. First and foremost, it is essential to acknowledge that not all anterior segment parameters provided by the two utilized devices were included in our analysis. Moreover, Scheimpflug imaging technology cannot optimally visualize chamber angle structures in details in compare to ultrasound devices and optical coherence tomography. In addition, measurements from topography systems employing different imaging modalities, such as slit scanning and optical biometry instrumentation, were not comparatively evaluated. Furthermore, it is imperative to emphasize that our investigation exclusively assessed healthy eyes. Consequently, there is a need for further research encompassing additional patient populations, including individuals with conditions such as corneal ectasia or corneal pathologies, who were intentionally excluded from the present study. The generalizability of our findings may be restricted to normal eyes, as we did not undertake a comprehensive analysis of diverse ocular conditions, which was beyond the scope of this research. Future studies should aim to conduct comparative analyses encompassing a wider range of devices and anterior segment parameters across various pathological groups to further investigate the reliability of the Scansys as an affordable anterior segment analyzer in comparison to the currently available similar technologies in the market.

## Conclusions

In the present investigation, for the first time, we conducted a comparative analysis of the anterior segment measurements between the Scansys and Sirius devices. We discovered that most outcomes obtained from both instruments were highly consistent and interchangeable, except for the anterior flat and steep keratometry, CCT and ACA. The results of the present study can support clinicians in their decision-making process when choosing between these techniques. Furthermore, the study can provide a framework for future studies that aim to compare the compatibility of different tomography devices.

## Data Availability

The datasets used and analyzed during the current study are available from the corresponding author on reasonable request.

## Abbreviations

ICC: Intraclass correlation coefficients
CCT: Central corneal thickness
TCT: Thinnest corneal thickness
ACD: Anterior chamber depth
ACA: Anterior chamber angle
HWTW: Horizontal white to white
